# Statistical data set for first-principles calculations of stacking fault energies in an AlNbTaTiV high entropy alloy

**DOI:** 10.1016/j.dib.2020.106670

**Published:** 2020-12-19

**Authors:** Joshua D. Strother, Chelsey Z. Hargather

**Affiliations:** Department of Materials Engineering, New Mexico Institute of Mining and Technology, 801 Leroy Place, Socorro, NM 87801 United States

**Keywords:** Body centered cubic, Stacking faults, Deformation, High entropy alloys, Density functional theory

## Abstract

High-entropy alloys (HEA), a new class of engineering alloy, are characterized by high concentrations of multiple main elements. These alloys have revealed a vast and largely unexplored compositional space that gives substantial promise for the discovery of new and interesting alloys and properties. In this data article, calculated data and applied inferential statistics are given for six structures related to the calculation of stacking fault energy in a refractory AlNbTaTiV BCC high-entropy alloy (HEA). Global populations of 120 atomic permutations of a special quasirandom structure are calculated for four of the six structures, and a complete statistical inference analysis is performed. Partial sample distributions are created for two of the six structures, and the trends and statistical parameters of the unknown global populations are predicted. The dataset refers to the research article “Stacking fault energies on the {112} planes of an AlNbTaTiV BCC high-entropy alloy from first-principles calculations, analyzed with inferential statistics” by Strother and Hargather [1].

## Specifications Table

**Table:** 

Subject	Materials Science
Specific subject area	Metals and Alloys
Type of data	Tables, Figures
How data was acquired	Density functional theory calculations using the commercial
	Vienna Ab-initio Simulation Package (VASP) code for electronic
	structure relaxations. For generating the HEA supercells,
	the Automated Theoretical Alloy Toolkit (ATAT) was used.
Data format	Raw data (tables), Analyzed data (figures and Supplementary Material)
Parameters for data collection	Electronic structure calculations of BCC supercells
	of layered {112} planes using: Projector augmented wave method,
	GGA-PBE pseudopotential, 0.1 meV/atom energy convergence,
	350 eV Plane-wave energy cutoff, and Blocked Davidson iterative
	scheme, and Methfessel-Paxton smearing for relaxations.
Description of data collection	Data were obtained from first-principles calculations
	performed using VASP v.5.4.1 on a Linux cluster running CentOS 7.
Data source location	New Mexico Institute of Mining and Technology
	Socorro, NM
	United State of America
Data accessibility	Within the article, Supplementary Material Excel file
Related Research Article	J. D. Strother, C. Z. Hargather, “Stacking fault energies on the {112}
	planes of an AlNbTaTiV BCC high-entropy alloy
	from first-principles calculations, analyzed with
	inferential statistics”, Materialia, 14, (2020) 100927.
	DOI: https://doi.org/10.1016/j.mtla.2020.100927

## Value of the Data

• Data are useful because it demonstrates a method for generating a database of materials properties based on statistical inference, with predictive error bars.
• Researchers in the field of materials science and engineering who do computational alloy design, ICME alloy design, or are interested in database generation will benefit from this data.
• The data can be reused to predict different sets of error bars and confidence intervals by using a different percentage of the global population. The method presented here can be applied to any other system for materials alloy design.
• The method and data presented in this work demonstrate a technique for rapid development of properties of high-entropy alloys.

## 1 Data Description

Six types of structures are investigated in the present work in a BCC AlNbTaTiV high-entropy alloy. This HEA is selected due to its equiatomic composition, solid solution BCC structure, presence of non-BCC elements, and non-magnetic elements. A defect free structure is investigated first. The five faulted structures studied are listed in the Table 1 below, and are described in detail in the research article that accompanies this data article [1]. Global populations that include the ground state energies of full sets of 120 atomic permutations are calculated for the NTS, T, and $TS_{1}$ faults. Partial populations of 30 and 20 atomic permutations of the ground state energy calculations are calculated for the $TS_{2}$ and $TS_{3}$ faults, respectively.

**Table 1:** The BCC (112) structures with their names [1] and # of atomic permutations calculated in the present work.

Name	# atomic permutations
Defect-free	120
NTS	120
$TS_{1}$	120
$TS_{2}$	30
$TS_{3}$	20
T	120

### 1.1 Raw data: Ground state energy calculations

The following table contains the raw data for the statistical analysis performed in the present work. Table 2 shows the 120 ground state energy calculations for each atomic permutation of the Defect-free, NTS, $TS_{1},$ and T structures in the AlNbTaTiV system. There are 30 ground state energy calculations for the $TS_{2}$ fault, and 20 ground state energy calculations for the $TS_{3}$ fault given in Table 2. Results are listed in no particular order for any of the faults.

**Table 2:** Raw data for the statistical inference analysis performed in the present work. Ground state energies of the various atomic permutations for the Defect-free, NTS,T,$TS_{1},$$TS_{2},$ and $TS_{3}$ structures in the AlNbTaTiV system, provided in eV/atom.

Defect-free (eV/atom)	NTS (eV/atom)	T (eV/atom)	$TS_{1}$ (eV/atom)	$TS_{2}$ (eV/atom)	$TS_{3}$ (eV/atom)
-8.5136	-8.5341	-8.5004	-8.5407	-8.5385	-8.5203
-8.5567	-8.5244	-8.5554	-8.5542	-8.5380	-8.5113
-8.5461	-8.5280	-8.5408	-8.5422	-8.5288	-8.5388
-8.5517	-8.5484	-8.5278	-8.5700	-8.5262	-8.5337
-8.5568	-8.5384	-8.5343	-8.5342	-8.5112	-8.5434
-8.5655	-8.5451	-8.5257	-8.5368	-8.5306	-8.5379
-8.5528	-8.5353	-8.5263	-8.5365	-8.5248	-8.5417
-8.5549	-8.5482	-8.5494	-8.5495	-8.5235	-8.5366
-8.5384	-8.5439	-8.5171	-8.5510	-8.5246	-8.5211
-8.5569	-8.5415	-8.5348	-8.5651	-8.5325	-8.5201
-8.5185	-8.5356	-8.5073	-8.5460	-8.5373	-8.5231
-8.5679	-8.5427	-8.5205	-8.5460	-8.5435	-8.5372
-8.5503	-8.5282	-8.5213	-8.5545	-8.5258	-8.5259
-8.5680	-8.5285	-8.5280	-8.5347	-8.5347	-8.5287
-8.5460	-8.5345	-8.5456	-8.5498	-8.5375	-8.5273
-8.5398	-8.5433	-8.5181	-8.5400	-8.5409	-8.5191
-8.5566	-8.5265	-8.5270	-8.5483	-8.5433	-8.5440
-8.5230	-8.5288	-8.5176	-8.5488	-8.5209	-8.5119
-8.5585	-8.5265	-8.5307	-8.5337	-8.5336	-8.5474
-8.5554	-8.5410	-8.5454	-8.5460	-8.5227	-8.5220
-8.5351	-8.5394	-8.5388	-8.5422	-8.5275	
-8.5618	-8.5453	-8.5421	-8.5488	-8.5335	
-8.5324	-8.5327	-8.5342	-8.5423	-8.5322	
-8.5504	-8.5402	-8.5147	-8.5469	-8.5274	
-8.5256	-8.5361	-8.5206	-8.5444	-8.5299	
-8.5648	-8.5334	-8.5252	-8.5723	-8.5215	
-8.5655	-8.5368	-8.5296	-8.5326	-8.5189	
-8.5329	-8.5262	-8.5368	-8.5580	-8.5249	
-8.5460	-8.5507	-8.5311	-8.5372	-8.5242	
-8.5286	-8.5255	-8.5297	-8.5465	-8.5212	
-8.5536	-8.5462	-8.5146	-8.5478		
-8.5614	-8.5446	-8.5479	-8.5578		
-8.5494	-8.5426	-8.5095	-8.5479		
-8.5590	-8.5388	-8.5168	-8.5434		
-8.5492	-8.5465	-8.5363	-8.5400		
-8.5640	-8.5388	-8.5298	-8.5410		
-8.5422	-8.5251	-8.5064	-8.5516		
-8.5649	-8.5499	-8.5308	-8.5477		
-8.5716	-8.5348	-8.5298	-8.5527		
-8.5291	-8.5431	-8.5306	-8.5449		
-8.5510	-8.5274	-8.5292	-8.5469		
-8.5508	-8.5449	-8.5294	-8.5536		
-8.5329	-8.5441	-8.5401	-8.5359		
-8.5526	-8.5432	-8.5305	-8.5458		
-8.5548	-8.5348	-8.5310	-8.5535		
-8.5623	-8.5444	-8.5358	-8.5421		
-8.5473	-8.5423	-8.5229	-8.5469		
-8.5547	-8.5335	-8.5228	-8.5524		
-8.5567	-8.5475	-8.5231	-8.5577		
-8.5591	-8.5460	-8.5461	-8.5434		
-8.5366	-8.5381	-8.5368	-8.5440		
-8.5478	-8.5417	-8.5332	-8.5370		
-8.5621	-8.5331	-8.5142	-8.5628		
-8.5384	-8.5345	-8.5332	-8.5365		
-8.5164	-8.5317	-8.5173	-8.5401		
-8.5542	-8.5451	-8.5242	-8.5454		
-8.5520	-8.5462	-8.5085	-8.5534		
-8.5689	-8.5254	-8.5451	-8.5359		
-8.5639	-8.5211	-8.5219	-8.5521		
-8.5522	-8.5478	-8.5196	-8.5401		
-8.5518	-8.5375	-8.5211	-8.5517		
-8.5208	-8.5444	-8.5395	-8.5304		
-8.5622	-8.5452	-8.5520	-8.5386		
-8.5590	-8.5356	-8.5198	-8.5506		
-8.5681	-8.5385	-8.5249	-8.5394		
-8.5534	-8.5510	-8.5302	-8.5492		
-8.5376	-8.5374	-8.5272	-8.5462		
-8.5518	-8.5493	-8.5085	-8.5590		
-8.5626	-8.5384	-8.5404	-8.5499		
-8.5582	-8.5392	-8.5238	-8.5496		
-8.5469	-8.5337	-8.5168	-8.5554		
-8.5116	-8.5299	-8.5367	-8.5525		
-8.5537	-8.5377	-8.5351	-8.5562		
-8.5654	-8.5362	-8.5336	-8.5357		
-8.5272	-8.5246	-8.5189	-8.5462		
-8.5675	-8.5312	-8.5097	-8.5503		
-8.5290	-8.5259	-8.5269	-8.5442		
-8.5271	-8.5360	-8.5371	-8.5419		
-8.5626	-8.5430	-8.5303	-8.5357		
-8.5669	-8.5338	-8.5054	-8.5501		
-8.5289	-8.5342	-8.5390	-8.5503		
-8.5683	-8.5266	-8.5241	-8.5472		
-8.5604	-8.5340	-8.5293	-8.5413		
-8.5335	-8.5236	-8.5235	-8.5474		
-8.5320	-8.5386	-8.5217	-8.5504		
-8.5626	-8.5334	-8.5373	-8.5400		
-8.5656	-8.5396	-8.5281	-8.5443		
-8.5645	-8.5414	-8.5060	-8.5397		
-8.5508	-8.5438	-8.5344	-8.5538		
-8.5511	-8.5318	-8.5276	-8.5474		
-8.5592	-8.5210	-8.5394	-8.5365		
-8.5509	-8.5350	-8.5232	-8.5540		
-8.5656	-8.5409	-8.5153	-8.5498		
-8.5713	-8.5470	-8.5306	-8.5449		
-8.5449	-8.5391	-8.5294	-8.5424		
-8.5492	-8.5366	-8.5291	-8.5594		
-8.5580	-8.5456	-8.5304	-8.5467		
-8.5344	-8.5465	-8.5381	-8.5576		
-8.5521	-8.5246	-8.5060	-8.5429		
-8.5442	-8.5366	-8.5323	-8.5490		
-8.5588	-8.5431	-8.5310	-8.5429		
-8.5488	-8.5324	-8.5270	-8.5423		
-8.5535	-8.5305	-8.5159	-8.5586		
-8.5532	-8.5581	-8.5254	-8.5440		
-8.5247	-8.5462	-8.5157	-8.5469		
-8.5313	-8.5270	-8.5100	-8.5579		
-8.5598	-8.5276	-8.5402	-8.5455		
-8.5420	-8.5314	-8.5260	-8.5448		
-8.5635	-8.5409	-8.5260	-8.5451		
-8.5561	-8.5339	-8.5127	-8.5457		
-8.5597	-8.5431	-8.5446	-8.5509		
-8.5603	-8.5345	-8.5227	-8.5604		
-8.5646	-8.5303	-8.5249	-8.5510		
-8.5661	-8.5433	-8.5315	-8.5460		
-8.5508	-8.5267	-8.5412	-8.5563		
-8.5239	-8.5456	-8.5432	-8.5413		
-8.5665	-8.5424	-8.5114	-8.5471		
-8.5646	-8.5471	-8.5387	-8.5386		
-8.5332	-8.5519	-8.5055	-8.5388		
-8.5298	-8.5318	-8.5065	-8.5450		

### 1.2 Analyzed data: Data sampling

**Fig. 1:**
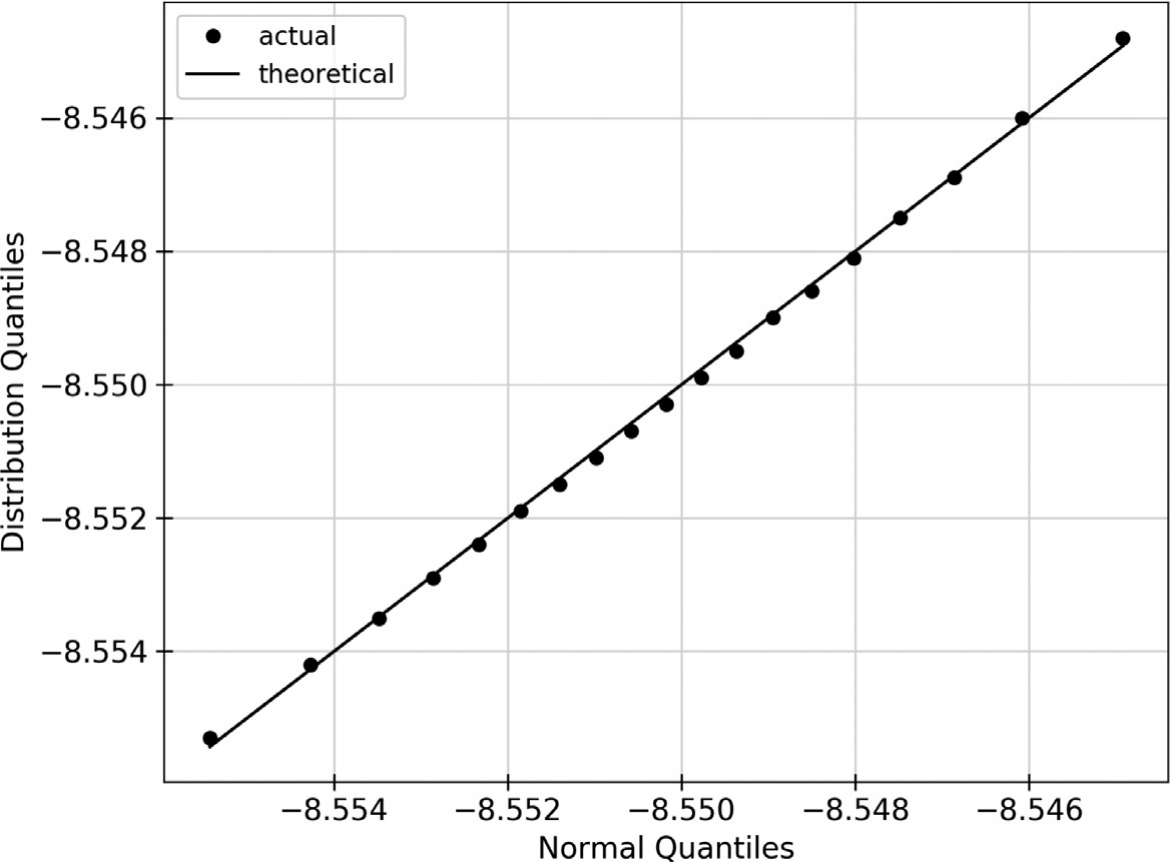
Inferential statistics analysis for a defect-free AlNbTaTiV BCC HEA cell showing the quantile versus quantile, Q-Q plot, where the black dots indicate the values for quantiles 0.1, 0.2, 0.3, *etc.* The black line shows the trend expected if the sampling distribution were normal.

Using the raw data given in Table 2, five types of analyzed data are presented for each of the structures listed in Table 1. The data in the following figures represents the random selection of the ground state energy of the atomic permutations, n, without replacement from a given faults’ population. Specific values relating to inferential statistics, defined below as (a) - (e), are calculated based on the random selection of the data from Table 2. In the case of the defect-free (Fig. 1) and $TS_{1}$ (Fig. 4) faults, some of the figures appear in the original research article that accompanies this data article [1] and they are not duplicated here.

(a) shows the sample mean, $\mu_{s},$ given in Eq. 3, as a function of the sample size, n, for an AlNbTaTiV SQS cell with no defects. As the ground state energy of each new atomic permutation is randomly drawn, $E_{0}$ is calculated and the mean is recalculated. Each dot on the figure represents the value of $\mu_{s},$ cumulative sample mean of $E_{0},$ for each value of n.
(b) shows the standard deviation of the sample, $\sigma_{s}$ as given in Eq. 4, as n increases for given structure. The ground state energy for each atomic permutations was randomly selected from the population without replacement, and the standard deviation was calculated. The standard deviation was recalculated for each n as each new $E_{0}$ value was added to the sample set.
(c) shows the standard sampling error, $\sigma_{m}$. This truncation error is effectively the expected error from considering only a sample of the total data set. This parameter is the standard deviation of the sampling distribution as discussed in Section 2.3. $\sigma_{m}$ can be calculated using two methods. The black dots indicate the predicted values while the blue line indicates the known values. The predicted values of $\sigma_{m}$ would be expected to converge closer to the known values as n increases. The value of $\sigma_{m}$ progresses towards and reaches zero as n=N. This effect is due to sampling without replacement. This is logical because there is no longer a truncation error if all data points have been considered.
(d) shows the sampling distribution of the mean with an n of 20. One million sample sets, m=1,000,000, are taken to construct the distribution, and each dot represents one of these sets. The one million average sample energies are then plotted on a frequency distribution plot. The vertical axis of the figure represents the number of sample energies that are equal to the energy listed on the horizontal axis. The black dots represent the sampling distribution. The solid green line shows a normal distribution overlay constructed with the same mean and standard deviation as the sampling distribution.
(e) shows a Q-Q plot for each of the distributions presented. Inferential statistics work for an n that is sufficiently large to make the sampling distribution normal. One common visual method of determining if a distribution is of a specific type is the quantile versus quantile, or Q-Q, plot. Quantiles are values along the range of a distribution where a certain percentage of the values will occur below the quantile value [2]. For example, a 0.1 quantile of 2 states that 10% of the probability distribution lies below the value of 2. The quantiles are calculated for the sampling distribution and an overlay normal distribution, then plotted against each other. The result should be linear with a slope of 1 if the sampling distribution can be considered normal. The black dots indicate the quantile values for 0.1, 0.2, 0.3, *etc*. The black line shows the expected trend if both distributions are perfectly normal.

The statistical analysis for the defect-free AlNbTaTiV BCC cell is given in Fig. 1 below and in Fig. 4 of the research article accompanying this work [1]. 120 atomic permutations of ground state energy calculations were performed on this cell. In [1], Fig. 4 gives (a) the sample mean, (b) the standard deviation, (c) the standard sampling error and (d) the sampling distribution of the mean. Below, Fig. 1 shows the Q-Q plot of the distribution which is a visual interpretation of the samples’ normality. The numerical values used to create this plot can be found in the Supplementary Material that accompanies this article.

**Fig. 2:**
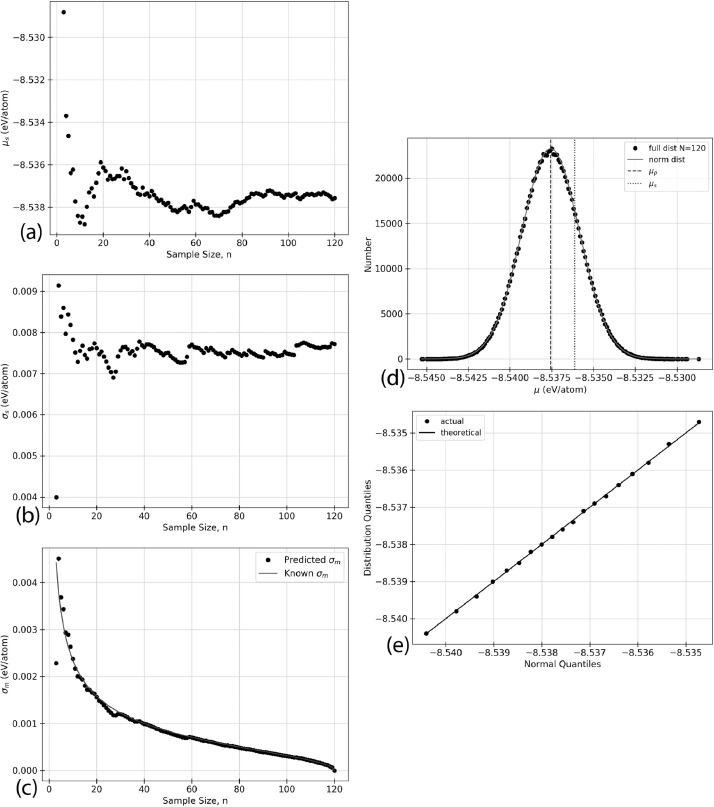
Inferential statistics analysis for a faulted NTS AlNbTaTiV BCC HEA cell showing (a) the mean cell energy, $E_{0},$ as a function of increasing sample size, n, (b) standard deviation, $\sigma_{s},$ as a function of increasing sample size, n, (c) standard sampling error, $\sigma_{m},$ as a function of increasing sample size, n, where $\sigma_{m}$ is given by the black dots, and he blue line indicates the known progression of $\sigma_{m}$ as calculated from $\sigma_{p}^{2},$ (d) the sampling distribution, where the black dots represent the sampling distribution, the solid green line represents a normal distribution with the same mean and standard deviation as the sampling distribution, the vertical dashed black line shows the mean of the population, $\mu_{p},$ and the vertical dotted blue line shows the mean of the sample, $\mu_{s},$ and (e) the quantile versus quantile, Q-Q plot, where the black dots indicate the values for quantiles 0.1, 0.2, 0.3, *etc.* The black line shows the trend expected if the sampling distribution were normal. (For interpretation of the references to colour in this figure legend, the reader is referred to the web version of this article.)

The statistical analysis for the faulted NTS AlNbTaTiV BCC cell is shown in Fig. 2. 120 atomic permutations ground state energy calculations were performed on this cell. Fig. 2 (a) shows the mean cell energy, $E_{0},$ as the sample size, n, increases from 3 to 120. Fig. 2 (b) shows the standard deviation of the sample as the sample size increases from 3 to 120. Fig. 2 (c) shows the standard sampling error as a function of increasing sample size from 3 to 120, which corresponds to the expected error bar from a given sample size, n. Fig. 2 (d) shows the sampling distribution of the mean with an n of 20 when 1,000,000 sample sets were taken. Finally, Fig. 2 (e) shows the Q-Q plot of the distribution which is a visual interpretation of the samples’ normality. The numerical values used to create these plots can be found in the Supplementary Material that accompanies this article.

**Fig. 3:**
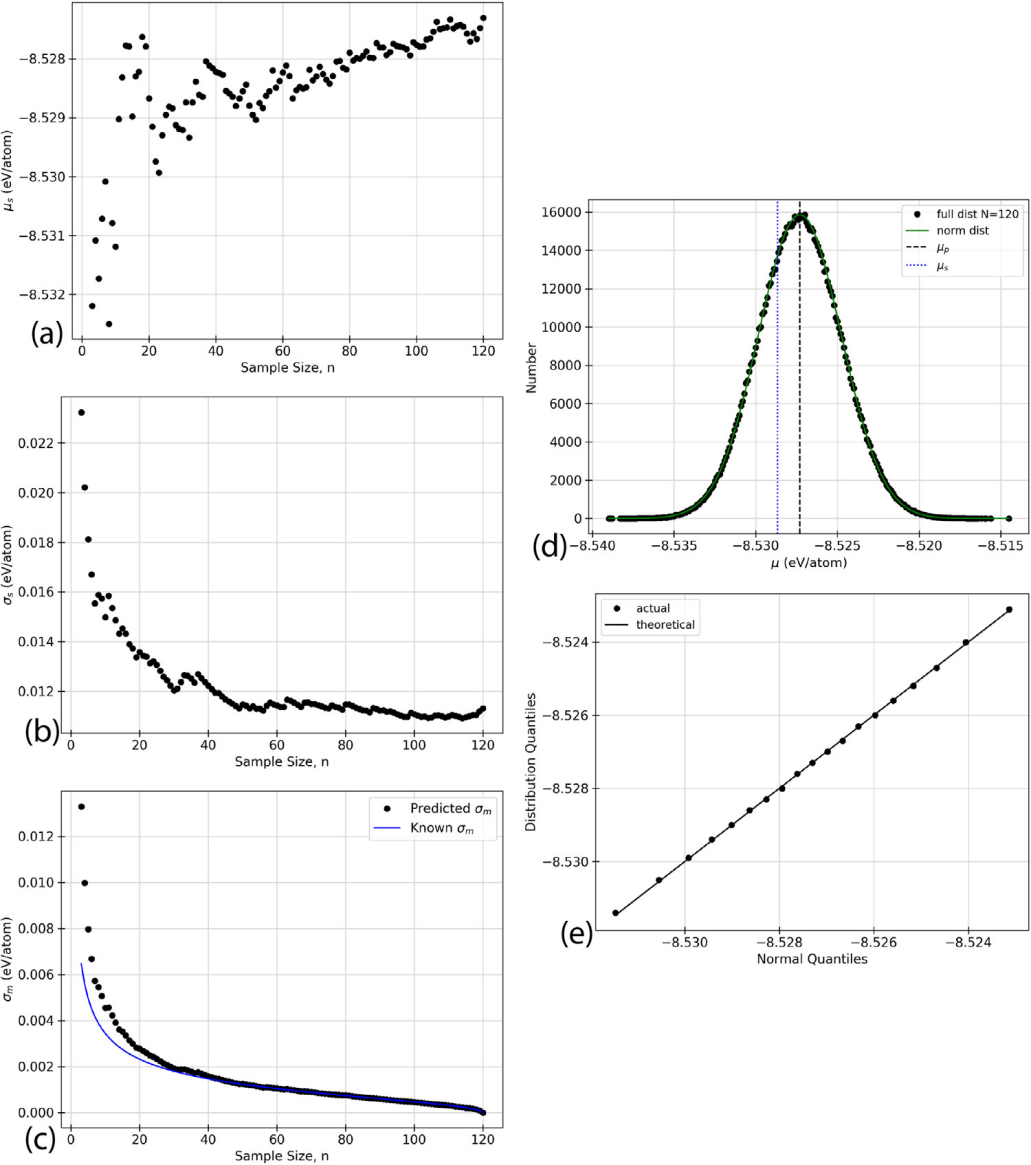
Inferential statistics analysis for a faulted T AlNbTaTiV BCC HEA cell showing (a) the mean cell energy, $E_{0},$ as a function of increasing sample size, n, (b) standard deviation, $\sigma_{s},$ as a function of increasing sample size, n, (c) standard sampling error, $\sigma_{m},$ as a function of increasing sample size, n, where $\sigma_{m}$ is given by the black dots, and the blue line indicates the known progression of $\sigma_{m}$ as calculated from $\sigma_{p}^{2},$ (d) the sampling distribution, where the black dots represent the sampling distribution, the solid green line represents a normal distribution with the same mean and standard deviation as the sampling distribution, the vertical dashed black line shows the mean of the population, $\mu_{p},$ and the vertical dotted blue line shows the mean of the sample, $\mu_{s},$ and (e) the quantile versus quantile, Q-Q plot, where the black dots indicate the values for quantiles 0.1, 0.2, 0.3, *etc.* The black line shows the trend expected if the sampling distribution were normal. (For interpretation of the references to colour in this figure legend, the reader is referred to the web version of this article.)

The statistical analysis for the faulted T AlNbTaTiV BCC cell is shown in Fig. 3. 120 atomic permutations ground state energy calculations were performed on this cell. Fig. 3 (a) shows the mean cell energy, $E_{0},$ as the sample size, n, increases from 3 to 120. Fig. 3 (b) shows the standard deviation of the sample as the sample size increases from 3 to 120. Fig. 3 (c) shows the standard sampling error as a function of increasing sample size from 3 to 120, which corresponds to the expected error bar from a given sample size, n. Fig. 3 (d) shows the sampling distribution of the mean with an n of 20 when 1,000,000 sample sets were taken. Finally, Fig. 3 (e) shows the Q-Q plot of the distribution which is a visual interpretation of the samples’ normality. The numerical values used to create these plots can be found in the Supplementary Material that accompanies this article.

**Fig. 4:**
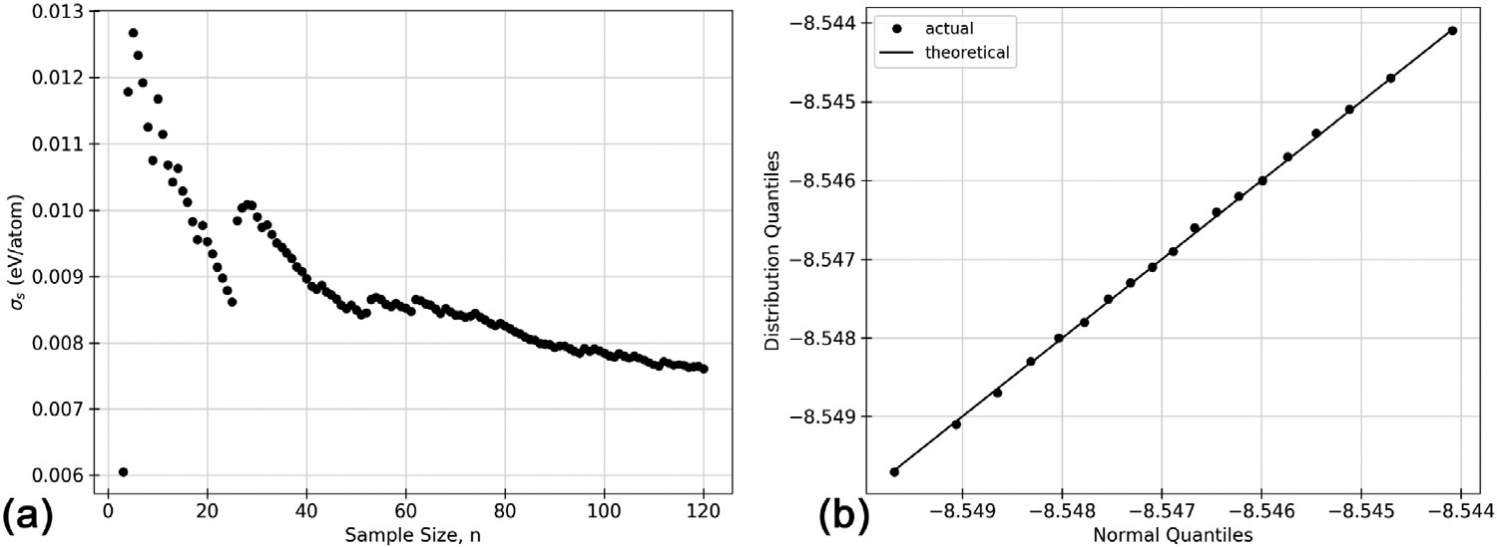
Inferential statistics analysis for a faulted $TS_{1}$ AlNbTaTiV BCC HEA cell showing (a) the sampling distribution, where the black dots represent the sampling distribution, the solid green line represents a normal distribution with the same mean and standard deviation as the sampling distribution, the vertical dashed black line shows the mean of the population, $\mu_{p},$ and the vertical dotted blue line shows the mean of the sample, $\mu_{s},$ and (b) the quantile versus quantile, Q-Q plot, where the black dots indicate the values for quantiles 0.1, 0.2, 0.3, *etc.* The black line shows the trend expected if the sampling distribution were normal. (For interpretation of the references to colour in this figure legend, the reader is referred to the web version of this article.)

**Fig. 5:**
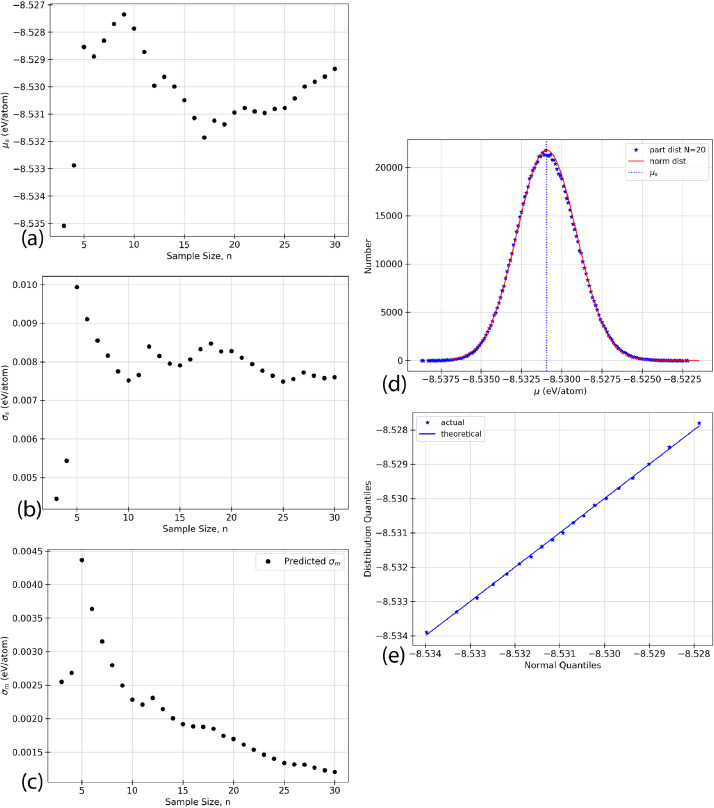
Inferential statistics analysis for a faulted $TS_{2}$ AlNbTaTiV BCC HEA cell showing (a) the mean cell energy, $E_{0},$ as a function of increasing sample size, n, (b) standard deviation, $\sigma_{s},$ as a function of increasing sample size, n, (c) standard sampling error, $\sigma_{m},$ as a function of increasing sample size, n, where $\sigma_{m}$ is given by the black dots, and the blue line indicates the known progression of $\sigma_{m}$ as calculated from $\sigma_{p}^{2},$ (d) the sampling distribution, where the black dots represent the sampling distribution, the solid green line represents a normal distribution with the same mean and standard deviation as the sampling distribution, the vertical dashed black line shows the mean of the population, $\mu_{p},$ and the vertical dotted blue line shows the mean of the sample, $\mu_{s},$ and (e) the quantile versus quantile, Q-Q plot, where the black dots indicate the values for quantiles 0.1, 0.2, 0.3, *etc.* The black line shows the trend expected if the sampling distribution were normal. (For interpretation of the references to colour in this figure legend, the reader is referred to the web version of this article.)

The statistical analysis for the faulted $TS_{1}$ AlNbTaTiV BCC cell is given in Fig. 4 below and in Fig. 5 of the research article accompanying this work [1]. 120 atomic permutations of ground state energy calculations were performed on this cell. In [1], Fig. 5 gives (a) the sample mean, (b) the standard sampling error, and (c) the sampling distribution of the mean. Below, Fig. 4 gives (a) shows the standard deviation of the sample as the sample size increases from 3 to 120, and (b) the Q-Q plot of the distribution which is a visual interpretation of the samples’ normality. The numerical values used to create these plots can be found in the Supplementary Material that accompanies this article.

The statistical analysis for the faulted $TS_{2}$ AlNbTaTiV BCC cell is shown in Fig. 5. 30 atomic permutations ground state energy calculations were performed on this cell and used to predict the properties of a global population set that would include 120 atomic permutations. Fig. 5 (a) shows the mean cell energy, $E_{0},$ as the sample size, n, increases from 3 to 30. Fig. 5 (b) shows the standard deviation of the sample as the sample size increases from 3 to 30. Fig. 5 (c) shows the standard sampling error as a function of increasing sample size from 3 to 30, which corresponds to the expected error bar from a given sample size, n. Fig. 5 (d) shows the partial sampling distribution of the mean with an n of 20 taken with replacement when 1,000,000 sample sets were taken. Finally, Fig. 5 (e) shows the Q-Q plot of the partial distribution which is a visual interpretation of the samples’ normality. The numerical values used to create these plots can be found in the Supplementary Material that accompanies this article.

**Fig. 6:**
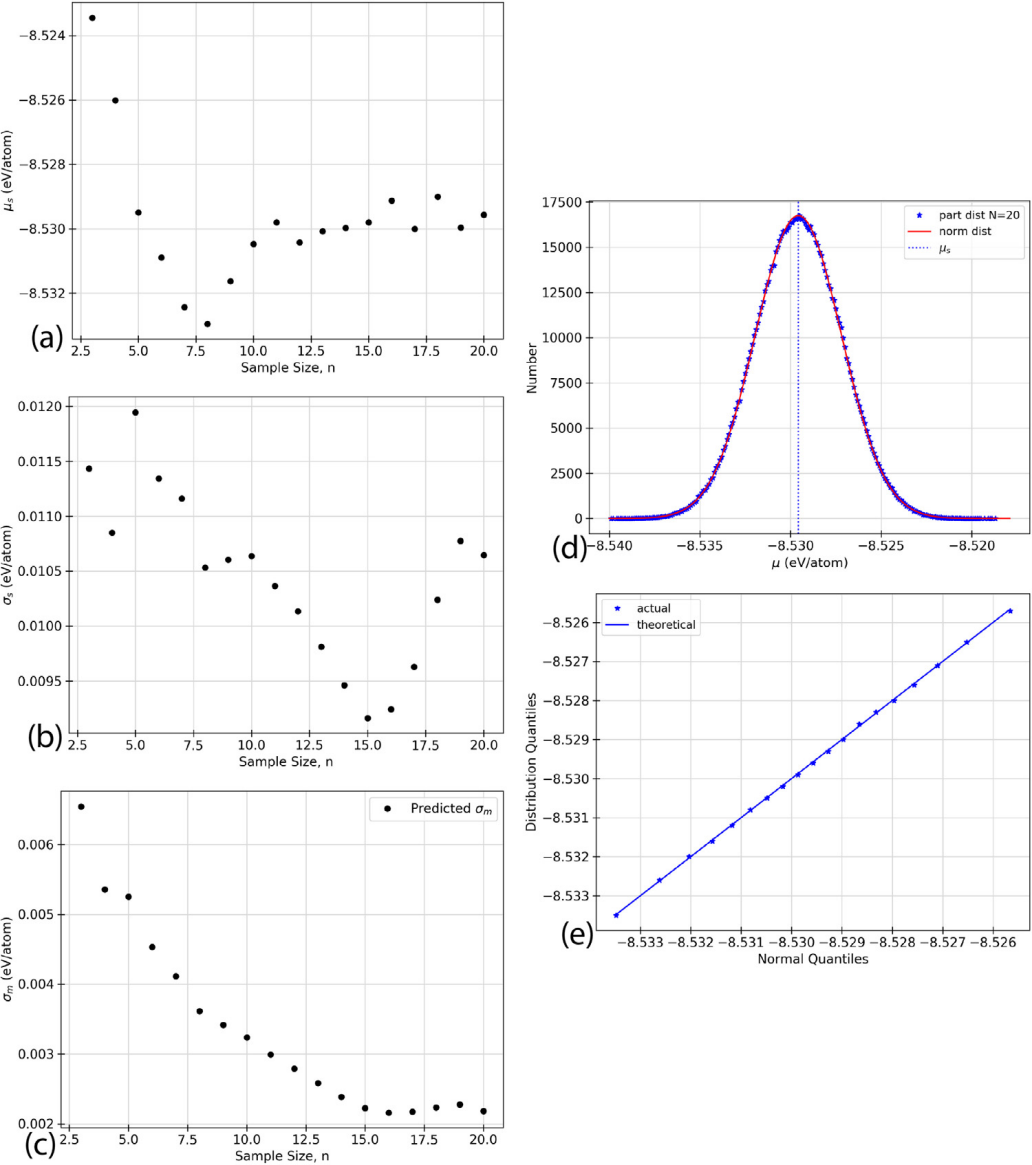
Inferential statistics analysis for a faulted $TS_{3}$ AlNbTaTiV BCC HEA cell showing (a) the mean cell energy, $E_{0},$ as a function of increasing sample size, n, (b) standard deviation, $\sigma_{s},$ as a function of increasing sample size, n, (c) standard sampling error, $\sigma_{m},$ as a function of increasing sample size, n, where $\sigma_{m}$ is given by the black dots, and the blue line indicates the known progression of $\sigma_{m}$ as calculated from $\sigma_{p}^{2},$ (d) the sampling distribution, where the black dots represent the sampling distribution, the solid green line represents a normal distribution with the same mean and standard deviation as the sampling distribution, the vertical dashed black line shows the mean of the population, $\mu_{p},$ and the vertical dotted blue line shows the mean of the sample, $\mu_{s},$ and (e) the quantile versus quantile, Q-Q plot, where the black dots indicate the values for quantiles 0.1, 0.2, 0.3, *etc.* The black line shows the trend expected if the sampling distribution were normal. (For interpretation of the references to colour in this figure legend, the reader is referred to the web version of this article.)

The statistical analysis for the faulted $TS_{3}$ AlNbTaTiV BCC cell is shown in Fig. 6. 20 atomic permutations ground state energy calculations were performed on this cell and used to predict the properties of a global population set that would include 120 atomic permutations. Fig. 6 (a) shows the mean cell energy, $E_{0},$ as the sample size, n, increases from 3 to 20. Fig. 6 (b) shows the standard deviation of the sample as the sample size increases from 3 to 20. Fig. 6 (c) shows the standard sampling error as a function of increasing sample size from 3 to 20, which corresponds to the expected error bar from a given sample size, n. Fig. 6 (d) shows the partial sampling distribution of the mean with an n of 20 taken with replacement when 1,000,000 sample sets were taken. Finally, Fig. 6 (e) shows the Q-Q plot of the partial distribution which is a visual interpretation of the samples’ normality. The numerical values used to create these plots can be found in the Supplementary Material that accompanies this article.

### 1.3 Skewness and kurtosis

To present a quantitative check that the sample populations are normal distribution and to prove that n values selected from Table 2 is sufficiently large for the images presented in Fig. 1 - Fig. 6, it is common is to look at the central moments of the distribution and compare them to those of a normal distribution. These moments are a measure of how the data are distributed around the mean. Table 3 shows these parameters for the full sampling distributions of the mean with n=20 for the defect free, NTS, T, and $TS_{1}$ structures. $TS_{2}$ and $TS_{3}$ are not included because full populations sets were not calculated. As can be seen in the table, the skewness and kurtosis values are all close to the values expected of a normal distribution, 0 and 3, respectively. Values within 0.1-0.2 of the ideal value are common for most normal distributions [2].

**Table 3:** The skewness and kurtosis parameters for the full sampling distributions of the mean for the AlNbTaTiV SFE calculations with n=20.

Method	Five Element	
Cell	Skewness	Kurtosis
112	0.17	2.98
NTS	0.02	2.97
T	0.03	2.98
$TS_{1}$	−0.11	3.03

## 2 Experimental Design, Materials and Methods

### 2.1 Density functional theory calculations

The Vienna *Ab initio* Simulation Package (VASP) was used for all DFT calculations [3], [4]. The projector augmented wave, PAW, pseudo-potentials with the generalized gradient approximation exchange correlation functional, GGA, as implemented by Perdew, Burke, and Ernzerhof were used for all calculations [5], [6], [7], [8]. All cells were converged to at least 0.1 meV/atom during relaxation. The cell shape and volume were constrained to prevent de-shearing of the faults during relaxation. Since the stacking fault structure differs from a defect-free structure by a shear displacement, the cell will de-shear during a full relaxation such as to produce a defect free structure and minimize the energy. In some cases, full relaxation was used, and is indicated in the text. A gamma centered k-mesh and reciprocal space projectors were used for all calculations [9]. Each k-mesh was at least 6x6x6. The fast Fourier transform mesh was set to contain all reciprocal vectors up to twice the largest basis vector. This mesh prevents wrap around errors during the fast Fourier transform [9]. A plane wave cut-off energy of 380 eV, 1.3 times the largest ENMAX value, was used for all calculations. The internal algorithm was set to the blocked Davidson iterative scheme [9]. The partial occupancy of orbitals was smeared with the Methfessel-Paxton first order method for relaxations and the tetrahedron method with Blöchl corrections for accurate total energy calculations [9].

### 2.2 Special quasi-random structures for stacking faults

Special quasi-random structures (SQS) are required for HEA calculations in DFT. These structures approximate a random solid solution with a given number of elements and their concentrations for a specific structural cell. An SQS cell must be generated for each structure, such as the different stacking fault cells listed in Table 1.

#### 2.2.1 Creation of special quasi-random structures

The Monte Carlo SQS (mcsqs) generation code from the Alloy Theoretic Automated Toolkit (ATAT) is used for all SQS cells generated in the present work [10]. mcsqs uses Monte Carlo simulations to search for a SQS that best represents a perfectly random structure [10]. The code randomly labels the atoms in a given cell structure and calculates the coordination parameters. It then compares the results to the theoretical coordination parameters for perfectly random structure [10]. Based on this comparison, the code identifies the best SQS structure found during the simulation using an objective function [10].

**Fig. 7:**
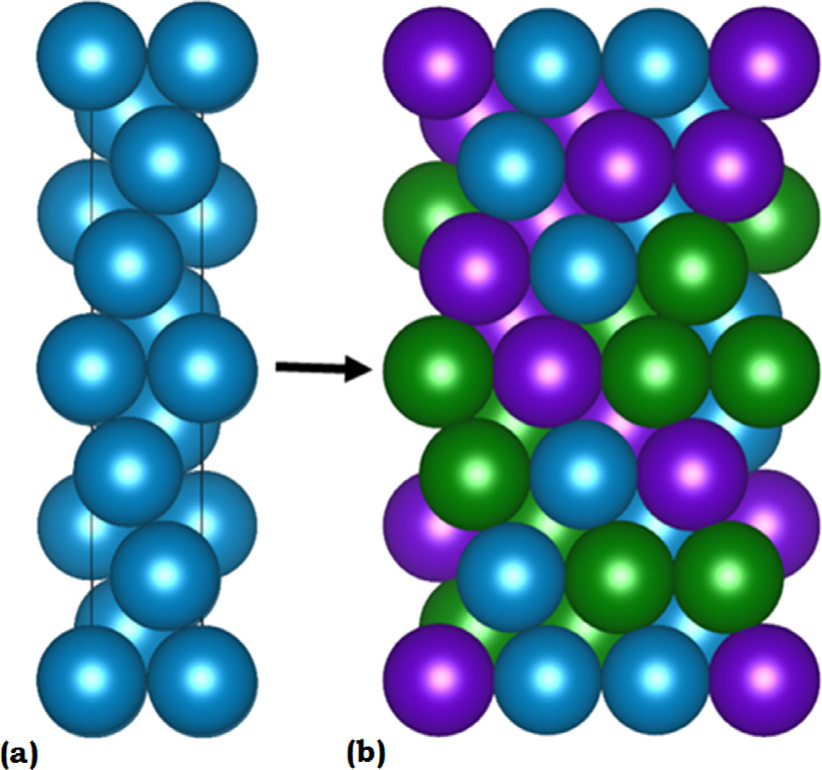
An input unit cell (a) for the SQS generation is shown on the left and fully defines the locations of the atoms but not the species. The output SQS cell (b) after generation is shown on the right. The size of the cell has been extended and all atomic species have been explicitly labeled, as indicated by the different colors.

The input cell structure for mcsqs is generally a unit cell that defines the structure parameters of the overall crystal [10]. The stacking fault cells need a unit cell that represents the stacking fault and the surrounding matrix. The simplest method of producing a unit cell for a stacking fault is to create a one atom wide stacking cell along the plane normal. Each atom plus the periodic boundaries fully represent a complete plane, the stacking plane in this case. Their relative positions along the cell axis represent the stacking sequence. For details on the faulted cells, see Fig. 1 and Fig. 2 in the research article that accompanies this data article [1]. During the SQS generation process, this unit cell is expanded parallel to the stacking plane to form the full supercell. Fig. 7 shows a standard set of input and output cells from an SQS generation. Fig. 7 (a) shows a unit cell defining the structure and location of atoms. Fig. 7 (b) shows a full SQS cell with increased size and explicitly labeled atomic species.

The final inputs for the mcsqs code are the correlation parameters to consider [10]. These parameters contain both the order and distance. The order refers to the number of atoms that are considered at a time. For instance, a second order correlation indicates how likely two atoms of specific species are to be near each other in the structure. The distance refers to the number of nearest neighbor shells that correlations are considered across. These settings were specified to consider at least second and third order correlations over at least three nearest neighbor shells for all SQS generations.

#### 2.2.2 Averaging of special quasirandom structure calculations

There is an error associated with using an SQS to represent a perfectly random solid solution [10]. There needs to be a metric for the quality of an SQS cell defined by how well it approximates a random structure. Such a metric can be developed by looking at the method used to generate an SQS. During generation, the cell is created to closely approximate the correlation parameters of a random alloy [10]. Fig. 7 (b) shows an example SQS cell with the atomic types marked by colors. A perfect SQS would be invariant with respect to atomic ordering. In other words, all atoms labeled “green” could be relabeled “purple” and all “purple” atoms labeled “green” without affecting the overall structure or its calculated ground-state energy. The quality of an SQS cell can be measured by calculating the energy associated with every permutation of atomic ordering and calculating the standard deviation of the energies [11]. This method provides a metric for how far the calculated ground state energy of the SQS can be expected to be from average.

Calculations averaged from different permutations of atomic assignments provide two useful insights into the system: an increase in accuracy to the calculated energy, and an insight into the sensitivity of the structure to variations in assignment. The first result has the benefit of increasing the fidelity of the calculations as a whole. The usefulness of the second result is more nuanced. For pure structure calculations, the standard deviation, or relative sensitivity, is simply a measure of the SQS quality [11]. For structures with a defect, this sensitivity is a superposition of both the SQS quality and the sensitivity of the defect to its surrounding atomic species [11]. This superposition makes it impossible to determine the percentage of the standard deviation that is attributed to each effect. Therefore, even if the SQS itself is perfect there will still be variations in the calculated energy due to the presence of a defect [11].

The calculations that required SQS cells were run with all possible permutations of atomic assignment. For an HEA of n equiatomic elements, there are n! possible permutations. A five element HEA has n!=120 permutations. For each structure, n! identical calculations were set up and each was assigned a unique cell from the list of possible atomic permutations. Afterwards, the n! energy values were collected, and the arithmetic mean and standard deviation were calculated from the set. Finally, the average energy value was used for further calculations, such as calculating SFE, and the standard deviation was stored to indicate the variation and approximate accuracy of the calculation.

### 2.3 Inferential statistics

The purpose of inferential statistics is to make inferences about a complete data set by examining a partial data set [2]. The primary usage of inferential statistics is an estimation of the variance of a sample parameter when compared to the known global parameter.

Inferential statistics are performed by sampling from a global population of N independent values, $x_{i},$ with i being an index. In the present work, $x_{i}$ are the ground state energy values represented in Table 2 for each of the different faulted structures. Each sample contains an independent and random selection of n values from the global population. m sample sets are then taken and used to produce a relative frequency distribution for a statistical parameter, such as mean or standard deviation. In this sampling distribution, each sample is used to produce one value. For example, a sampling distribution of the means is a distribution produced by creating the frequency diagram from the m sample means. This sampling procedure produces an effect on the data given by the central limit theorem [2]. The central limit theorem states that as the sample size increases, the sampling distribution of the mean approaches a normal distribution. The sampling distribution has the same mean as the original population and a variance of $\frac{\sigma^{2}}{n},$ where $\sigma^{2}$ is the population variance and n is the sample size [2]. Effectively, as n increases, the frequency distribution of the sample means converges towards the population mean and takes the shape of a normal distribution with a converging variance. The properties of a normal distribution can be attributed to the sampling distribution regardless of the shape of the global distribution [2].

There is an important requirement for this statistical analysis. The value of n must be sufficiently large. The main reason is that the average sample must reasonably represent the original population. A single value can vary drastically from the mean and contains no information on population variance. However, as more values are added, the sample mean and variance become estimators for the for the total population mean and variance. As n becomes large enough, the central limit theorem ensures the sampling distribution becomes normal. Therefore, a normally distributed sampling distribution can be used to prove that the value of n was sufficient.

In this data article, the sampling distribution of the mean will be used to estimate the global mean and the expected variance of the sample means from the global mean. For SFE calculations in HEA systems, the desired cell ground state energy value is determined to be the mean from all possible atomic permutations. The important parameters of the global population are the population size, the mean, and the variance. The population mean, $\mu_{p},$ is calculated in Eq. 1:

(1) $$\mu_{p}=\frac{\sum_{i=0}^{N}x_{i}}{N}$$

where N and $x_{i}$ are as described above. The population variance, $\sigma_{p}^{2}$, is calculated as shown in Eq. 2 [2]:

(2) $$\sigma_{p}^{2}=\frac{\sum_{i=0}^{N}\left(x_{i}-\mu_{p}\right){}^{2}}{N}$$

A sample of size n permutations is selected from the calculated cell energies, $x_{i},$ given in Table 2. The sample mean, $\mu_{s},$ is calculated by Eq. 3 [2]:

(3) $$\mu_{s}=\frac{\sum_{i=0}^{n}x_{i}}{n}$$

The variance of the sample population, $\sigma_{s}^{2},$ is calculated as shown in Eq. 4 [2]:

(4) $$\sigma_{s}^{2}=\frac{\sum_{i=0}^{n}\left(x_{i}-\mu_{s}\right){}^{2}}{n}$$

The mean of the m=1,000,000 sampling means, $\mu_{m}$ is calculated as shown in Eq. 5:

(5) $$\mu_{m}=\frac{\sum_{i=0}^{m}\mu_{si}}{m}$$

The mean of the sampling means is the average mean obtained during sampling. The variance of the sampling means without replacement, $\sigma_{m}^{2}$ is calculated in Eq. 6:

(6) $$\sigma_{m}^{2}=\frac{\sigma_{p}^{2}}{n}\frac{\left(N-n\right)}{\left(N-1\right)}$$

The variance of the sampling distribution without replacement when $\sigma_{p}^{2}$ is not known is written as:

(7) $$\sigma_{m}^{2}\approx \frac{s^{2}}{n}\frac{\left(N-n\right)}{\left(N-1\right)}$$

The central theorem also provides a relationship between $\sigma_{m}^{2}$ and $\sigma_{p}^{2}$ [2]. This relation is derived from the variance sum law [2] and given in Eq. 8:

(8) $$\sigma_{m}^{2}=\frac{\sigma_{p}^{2}}{n}.$$

The primary interest of the statistical analysis performed herein is in the relationship between the population and sample parameters, which compares $\mu_{s}$ to $\mu_{p}$ and $\sigma_{s}^{2}$ to $\sigma_{p}^{2}$. The sample mean converges towards the population mean as n increases [2]. $\sigma_{s}^{2}$ also converges to $\sigma_{p}^{2}$ as n increases. The final relation of interest is between the population variance and the variance of the sample means which can be derived from the variance sum law and is stated in the central limit theorem [2]. The variance of the sample means, $\sigma_{m}^{2},$ converges to $\frac{\sigma_{p}^{2}}{n}$ as n increases. $\sigma_{p}^{2}$ is not a known value if only one sample has been taken. Since $\sigma_{p}^{2}$ is not known, an estimator for $\sigma_{p}^{2}$ must be used. An unbiased estimator for the population variance is the sample variance estimator, $s^{2}$ [2]. This parameter is calculated as:

(9) $$s^{2}=\frac{\sum_{i=0}^{n}\left(x_{i}-\mu_{s}\right){}^{2}}{n-1}$$

Note there is a n−1 in the denominator instead of the familiar n [2]. The n−1 in the denominator makes it an unbiased estimator due to the degrees of freedom [2]. Substituting $s^{2}$ for $\sigma_{p}^{2}$ in Eq. 8 yields the following relation:

(10) $$\sigma_{m}^{2}\approx \frac{s^{2}}{n}$$

for large n [2].

The variance of the sample means, $\sigma_{m}^{2},$ is important, as it is the metric for how far from $\mu_{p}$ the $\mu_{s},$ of n values is expected to vary [2]. Essentially, $\sigma_{m}^{2}$ is a predicted truncation error. This truncation error is the difference between the mean of the total population and the mean of the sample. A more intuitive form of this error is the standard sampling error which is the square root of the variance and is given as:

(11) $$\sigma_{m}\approx \sqrt{\frac{s^{2}}{n}}=\frac{s}{\sqrt{n}}$$

with $\sigma_{m}$ being the standard sampling error [2]. This is also called the standard error of the mean and is more intuitive as it has the same units as the x values [2]. Since the sampling distribution is a normal distribution the normal sigma probabilities apply [2]. As such, 95% of all samples will lie within bounds of twice the SSE from the mean, or 2-sigma [2].

#### 2.3.1 Sampling without replacement

In Section 2.3, sampling with replacement from a global population was discussed. This means that each value selected was truly random and independent of previous selections. In DFT calculations, sampling from Table 2 without replacement is desired. Sampling without replacement means that each value is randomly selected and then withheld from the population for the next selection and so forth. Sampling without replacement, from a finite population, leads to a negative correlation between the values already selected and the next value to be selected [2]. If an especially large value is selected and removed from the available dataset, the next value will be more likely to be smaller and a negative correlation results. This correlation has an effect on the variance of the sampling distribution and a correction factor is required. The new equation for calculating the $\sigma_{m}^{2}$ value, when $\sigma_{p}^{2}$ is known, is given as:

(12) $$\sigma_{m}^{2}=\frac{\sigma_{p}^{2}}{n}\left(\frac{N-n}{N-1}\right).$$

The variance of the sampling distribution when $\sigma_{p}^{2}$ is not known is written as:

(13) $$\sigma_{m}^{2}\approx \frac{s^{2}}{n}\left(\frac{N-n}{N-1}\right).$$

These equations are for sampling without replacement [2]. All other variables remain the same. This method of sampling and the resulting equation has an immediate benefit. The value of $\sigma_{m}^{2}$ goes to zero as n goes to N when sampling without replacement. Another benefit of sampling without replacement is a consistent number of calculations. These statistics will be used to calculate the estimated statistical parameters from a sample of the total calculations. For a sampling procedure with sample size n, only n atomic permutations will be randomly selected, ensuring a consistent number of calculations.

#### 2.3.2 Skewness and kurtosis

Skewness and kurtosis are are a measure of how the data from a particular set are distributed around the mean. The third and fourth central moments are commonly called skewness and kurtosis, respectively. The skewness represents the symmetric nature of the tails of the distribution and is zero for a perfectly normal distribution. Eq. 14 shows how skewness is calculated:

(14) $$skewness=\sum_{i}^{N}\frac{\left(x_{i}-\mu_{z}\right){}^{3}}{\left(N*\sigma_{z}\right){}^{3}}$$

where the subscript z represents the sample population (z=s) or the global population (z=p). N is the total number of data points being considered in the calculation.

Kurtosis is defined as the fourth central moment of the population distributed around the mean. The kurtosis represents the relative weight of the tails versus the center of the distribution and equals three for a normal distribution. Eq. 15 shows how kurtosis was calculated in the present work:

(15) $$kurtosis=\sum_{i}^{N}\frac{\left(x_{i}-\mu_{z}\right){}^{4}}{\left(N*\sigma_{z}\right){}^{4}}$$

where the subscript z represents the sample population (z=s) or the global population (z=p). N is the total number of data points being considered in the calculation.

## CRediT Author Statement

**Joshua D. Strother:** Conceptualization, Methodology, Investigation, Formal analysis, Writing review & editing. **Chelsey Z. Hargather:** Conceptualization, Funding acquisition, Resources, Supervision, Writing original draft

## Declaration of Competing Interest

The authors declare that they have no known competing financial interests or personal relationships which have, or could be perceived to have, influenced the work reported in this article.

## Appendix A Supplementary materials

Supplementary Data S1 Supplementary Raw Research Data. This is open data under the CC BY license http://creativecommons.org/licenses/by/4.0/ Supplementary Data S1
